# Extracting the winter wheat using the decision tree based on time series dual-polarization SAR feature and NDVI

**DOI:** 10.1371/journal.pone.0302882

**Published:** 2024-05-08

**Authors:** Huiyang Zhang, Zhiyong Wang, Zhenjin Li, Xiaotong Liu, Kai Wang, Shichang Sun, Silong Cheng, Zhenhai Gao

**Affiliations:** 1 College of Geodesy and Geomatics, Shandong University of Science and Technology, Qingdao, China; 2 National Demonstration Center for Experimental Surveying and Mapping Education (Shandong University of Science and Technology), Qingdao, China; USACE ERDC: US Army Engineer Research and Development Center, UNITED STATES

## Abstract

Winter wheat is one of the most important crops in the world. It is great significance to obtain the planting area of winter wheat timely and accurately for formulating agricultural policies. Due to the limited resolution of single SAR data and the susceptibility of single optical data to weather conditions, it is difficult to accurately obtain the planting area of winter wheat using only SAR or optical data. To solve the problem of low accuracy of winter wheat extraction only using optical or SAR images, a decision tree classification method combining time series SAR backscattering feature and NDVI (Normalized Difference Vegetation Index) was constructed in this paper. By synergy using of SAR and optical data can compensate for their respective shortcomings. First, winter wheat was distinguished from other vegetation by NDVI at the maturity stage, and then it was extracted by SAR backscattering feature. This approach facilitates the semi-automated extraction of winter wheat. Taking Yucheng City of Shandong Province as study area, 9 Sentinel-1 images and one Sentinel-2 image were taken as the data sources, and the spatial distribution of winter wheat in 2022 was obtained. The results indicate that the overall accuracy (OA) and kappa coefficient (Kappa) of the proposed method are 96.10% and 0.94, respectively. Compared with the supervised classification of multi-temporal composite pseudocolor image and single Sentinel-2 image using Support Vector Machine (SVM) classifier, the OA are improved by 10.69% and 5.66%, respectively. Compared with using only SAR feature for decision tree classification, the producer accuracy (PA) and user accuracy (UA) for extracting the winter wheat are improved by 3.08% and 8.25%, respectively. The method proposed in this paper is rapid and accurate, and provide a new technical method for extracting winter wheat.

## 1 Introduction

As the global population continues to grow, the demand for food is also increasing. Wheat, as one of the most widely grown crops in the world, accounts for approximately 20% of human energy consumption and plays a crucial role in the food supply [1]. As the world’s leading wheat producer and consumer [2], China places paramount importance on the precise and timely monitoring of wheat planting distribution. This critical task is instrumental in informing the nation’s economic development strategies, regulating its planting structure, and safeguarding food security [3–5]. Traditional agricultural statistical reports and sampling survey methods are not able to quickly and accurately obtain crop planting distribution information [4]. This process not only consumes a considerable amount of manpower and resource but also leads to unpredictable errors [6,7]. With the development of remote sensing technology, it has made it possible to quickly and accurately obtain information on the area and distribution of crop cultivation [8], which is more economical and efficient compared with traditional field survey methods [9–11].

Optical and SAR images are the main data for winter wheat extraction. The optical images contain rich spectral information, and special ground object can be extracted effectively by band math. Among them, the extraction of the NDVI is currently considered the best indicator for detecting vegetation growth status [12]. For example, Zhang Huanxue et al. (2015) [13] used HJ-1 A/B CCD satellite data to construct NDVI time series profile. They used decision tree classification method at the object scale to classify wheat, corn, rice, soybean, and potato in Hongxing Farm, Heilongjiang Province, achieving the OA of 92.2%. Aleem Khaliq (2018) et al. [14] extracted NDVI based on Sentinel-2 and selected the best phenological features to input the random forest classifier to classify crops in the Italian region. Xu Feng et al. (2020) [15] extracted winter wheat in Shandong Province based on the Google Earth Engine (GEE) by extracting NDVI as the feature to input random forest classifier, with the OA of 93.4%. Many scholars have demonstrated that the NDVI can be used for monitoring crop [12–15]. However, although optical images are highly accurate, they are easily affected by the problems such as lighting condition, cloud cover, and rain [10]. It means that adequate time series images can’t be obtained. Due to the small number of optical images, the extraction results are more likely to be affected by abnormal pixel values or noise, with a certain degree of contingency, and cannot meet the requirements of area estimation in units of years [10].

Synthetic Aperture Radar (SAR), as an active imaging radar, is not affected by lighting, cloud, or rain and it has the advantages of day/night images acquisition and all-weather imaging capability [16]. Therefore, it can provide sufficient time series images for monitoring and extracting the winter wheat [17]. As the most typical feature of SAR, backscattering coefficient has achieved promising results in the field of winter wheat monitoring. For instance, Rao Zahid Khalil et al. (2017) [18] extracted the mean backscattering coefficient from Sentinel-1 data and used the maximum likelihood classifier to classify ground object in the Okara region of Pakistan with the OA reaching 80%. Song Yang et al. (2019) [19] used time series Sentinel-1 data to extract the backscattering coefficient under VH and VV polarization. They used the parallel hexahedron classifier to map the winter wheat planting for the central part of the North China Plain in China with the OA reaching 84%. Michael Schlund et al. (2020) [20] used time series Sentinel-1 data to extract the backscattering coefficient under VH and VV polarization of winter wheat from 2017–2019 in an agricultural region in Germany and performed band math to successfully monitor the phenological period of winter wheat. The minimum monitoring error for phenological periods of winter wheat is 5 days in 2017. Rouhollah et al. (2021) [21] used Sentinel-1 data to extract the backscattering coefficient as monitoring tool for crop growth and successfully achieved phenological monitoring of wheat on a farm in Turkey. The experimental results indicate that the multi temporal SAR backscattering coefficient provides more detailed information for monitoring the entire growth stage of crops. Mutasha Brian Mulenga et al. (2022) [22] extracted the mean backscattering coefficient of wheat at different growth stages from time series Sentinel-1 data and used the Support Vector Machine (SVM) classifier to extract wheat in the Philippines. The OA can reach 80.6%. Many scholars have demonstrated that SAR backscattering coefficient have achieved good results in crop monitoring and extraction. However, the inherent noise in the SAR backscattering coefficient also affects the accuracy of ground target monitoring and extraction, leading to difficulties in extracting crop with high precision. Therefore, some scholars [18,22] in the above studies used mean backscattering coefficient to limit the effect of noise on the results, which also provided a reference for our research.

In general, SAR and optical images have their own advantages and disadvantages. They can complement each other through synergy. Therefore, it is feasible to improve the accuracy of crop monitoring by combing SAR and optical data. Some scholars have conducted research on crop classification and recognition based on the combination of SAR and optical data, and the results indicate that the classification accuracy achieved through multi-sensor fusion is generally higher than that of single sensor [23,24]. Zhou Tao et al. (2017) [25] collaborated to use Sentinel-1 and Landsat-8 data to extract texture features and backscattering coefficient from SAR data and NDVI from optical data. These features are used to input the SVM classifier, successfully enabling the extraction and mapping of winter wheat in Gaochun District, Nanjing City, China. The OA reached 98.06%. Cai Yaotao et al. (2019) [26] used Sentinel-1 data along with combination of MODIS and Sentinel-2 data. They extracted time series backscattering coefficient and time series NDVI. After selecting the best features combination, they successfully used the random forest classifier to extract and map the rice in the Dongting Lake region of China with the OA of 95%. Wang Limei et al. (2022) [27] proposed an object-based method for automatic identification of winter wheat based on the fusion of time series Sentinel-1 and Sentinel-2 data. The OA reached 92%, and the Kappa coefficient was 0.84. Wu Wenfu et al. (2022) [28] have explored the fusion of multispectral and SAR features with machine learning algorithms. The research results indicate that the fusion of SAR and optical images have a higher leniency for registration errors. Machine learning algorithms have been widely applied with a high level of confidence in tasks such as classification and regression. Fu Bolin et al. (2023) [29] have achieved the classification of mangrove species by combing multidimensional optical and SAR images with machine learning. The OA increased by 12.85%. In addition, the combined use of optical and SAR data has achieved good results in other cases such as wetland classification [30].

Obviously, in the application of combining SAR and optical features for ground extraction [25–27], the most methods input features into classifiers and select samples for supervised classification. For information such as texture features derived from single SAR image, the discriminative capability is usually limited by the resolution and noise constraints of the original image, and it takes a long time to input the features into some typical machine learning classifiers such as RF and KNN. Recently, the identification of winter wheat planting area is of great concern. Winter wheat, as an important food crop, is widely planted and consumed worldwide, which is crucial for ensuring food supply and meeting people’s living needs. Therefore, accurately identifying the planting area of winter wheat is crucial for achieving efficient management and resource allocation of grain production. In winter wheat monitoring, the area estimation is usually carried out for a city, and the study area is large, so the classification method is required to consider the efficiency while ensuring the accuracy. For a long time, there is a lack of a fast and accurate method for monitoring the area of winter wheat. When contrasted with deep learning algorithms, machine learning algorithms demonstrate superior accuracy when working with limited samples and produce results that are interpretable, highlighting a key advantage of machine learning algorithms and the machine learning algorithms training speed is fast and suitable for scenarios with high real time requirements. Decision Tree Classification (DTC) [31] classifies each pixel in the image sequentially by dividing it with threshold values. It runs quickly and avoids misclassification and omission caused by sample selection limitation, making it substantially advantageous in ground classification [32]. By combining the advantages of SAR and optical images, we have made up for their respective shortcomings. In this paper, we construct a fast and relatively accurate decision tree model for winter wheat extraction by properly processing the backscattering coefficient and combining with the NDVI. The study is conducted in Yucheng City, Shandong Province, China, and the precise mapping of winter wheat is successfully achieved.

The structure of this paper is as follows: Section 2 presents the study area and data. In section 3 the research method used in this study is presented, including the generation and processing of dual-polarization backscattering coefficient and NDVI and the construction of the decision tree. Section 4 presents the experimental classification results of this study and compares them with the results of SVM classification using multi-temporal composite SAR pseudocolor image and single Sentinel-2 image. It also discusses and explores different methods of combining SAR feature and NDVI. In section 5, we provide a qualitative evaluation of spatial details for decision tree method proposed in this paper and SVM method. In the end presents some important conclusions obtained from the study.

## 2 Materials and methods

### 2.1 Study area

**Fig 1:**
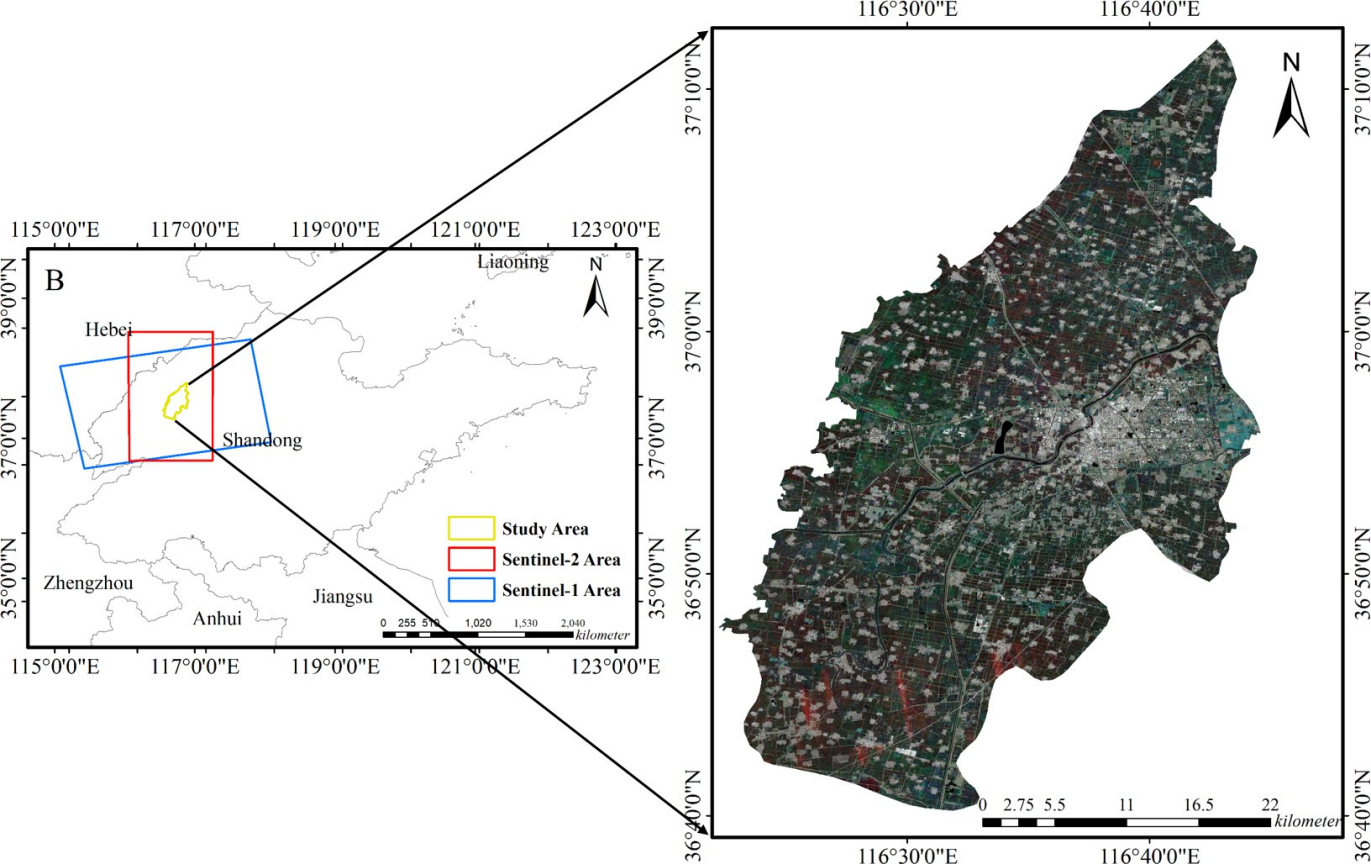
Study area map. The yellow box represents the study area of this paper, the blue box represents the coverage of Sentinel-1 data from the ascending orbit, and the red box represents the coverage of Sentinel-2 data.

Yucheng city is located in the northwest of Shandong Province, China. It has a total area of 990km^2^ belonging to the alluvial plain of the lower reaches of the Yellow River. It latitude and longitude coordinates are 36°41′-37°12′N, 116°22′-116°45′E and it elevation ranges from 17.5m to 26.1m with less topographical ups and downs. It has a temperate continental monsoon climate, with four distinct seasons and obvious dry and wet seasons, showing very obvious winter cold and summer heat characteristics. The average annual temperature is 13.3°C, the annual sunshine is 2546.2h, the average annual precipitation is 555.5mm, the average annual evaporation is 1884.8mm, and the average annual frost-free period is 202d, which is very suitable for the planting of winter wheat. Winter wheat is usually sown in October of the previous year and harvested in early June of that year. According to the information released by the Ministry of Agriculture and Rural Affairs of the People’s Republic of China, the phenological stages of winter wheat in Yucheng can be divided into some stages: regreening, tillering, overwintering, regreening, erecting, jointing, booting, heading, flowering, filling, waxing, maturing. The geographical extent of Yucheng is shown in Fig 1. In addition to winter wheat, typical ground objects in the study area includes building, water, and other vegetation. The information is sourced from the public website of the research area government (www.yuchengshi.gov.cn).

### 2.2 Data

#### 2.2.1 Satellite data

We obtained a total of 9 Sentinel-1A GRD dual-polarization data from March to June 2022, covering the winter wheat growing period from regreening to maturity. These data are used to extract the SAR backscattering coefficient under VH and VV polarization. Using A-I to represent 9 different phenological periods of winter wheat (from regreening to maturity). The phenological period and crop calendar corresponding to the images are shown in Table 1.

**Table 1:** Winter wheat growth period and SAR image parameters.

Lable	Phenological cycle	Date	Orbit Direction	Polarization	Incidence angle(°)	Path
**A**	regreening	March 7th	Ascending	VH/VV	38.9	142
**B**	erecting	March 19th	Ascending	VH/VV	38.9	142
**C**	jointing	March 31th	Ascending	VH/VV	38.9	142
**D**	booting	April 12th	Ascending	VH/VV	38.9	142
**E**	heading	April 24th	Ascending	VH/VV	38.9	142
**F**	flowering	May 6th	Ascending	VH/VV	38.9	142
**G**	seeding	May 18th	Ascending	VH/VV	38.9	142
**H**	waxing	May 30th	Ascending	VH/VV	38.9	142
**I**	maturing	June 11th	Ascending	VH/VV	38.9	142

A-I represents the nine growth stages of winter wheat and their corresponding images acquisition times.

Sentinel-1 is an Earth observation satellite within the Copernicus Program of the European Space Agency (ESA). It is part of the global environmental and security monitoring system initiated by the European Commission (EC) and the European Space Agency [5]. There are two polar-orbiting satellites, both equipped with C-band sensors, with a revisit cycle of 12 days. The imaging mode of all data are IW (Interferometric Wide), VH/VV dual-polarization, ascending orbit, and the incidence angle is about 38.9°. The SAR image parameters used in this study are shown in Table 1. The all Sentinel-1 images are downloaded from the ESA (https://dataspace.copernicus.eu/).

The Sentinel-2 data aquired during the winter wheat maturity stage was used to extract vegetation index feature, with an imaging date of June 8, 2022. Sentinel-2 is a satellite imaging mission carried out as part of the Copernicus Programme by the European Commission and the European Space Agency [5], which has a total of 13 multispectral bands [33].

#### 2.2.2 Sample and statistical yearbook data

The sample data are divided into four categories: winter wheat, water, building, and other vegetation. The region of interest in the study area are selected based on Google Earth image, Sentienl-2 image, and the results of other study [34]. The samples are randomly divided into training samples and validation samples. 70% of the samples are allocated for model construction, and remaining 30% are reserved for assessing classification accuracy. The quantities of sample data are shown in Table 2.

**Table 2:** Number of samples.

Type	Winter Wheat	Other Vegetation	Building	Water	Total
**Training**	42	21	42	21	126
**Validation**	18	9	18	9	54

A statistical yearbook is a large-scale tool that records annual economic, social and other developments in a comprehensive, systematic and continuous manner through highly dense statistical data, mainly in the form of statistical charts and analytical descriptions. The winter wheat planting area of study area in the statistical yearbook data is 497km^2^, obtained from the official website (www.yuchengshi.gov.cn.).

## 3 Method

**Fig 2:**
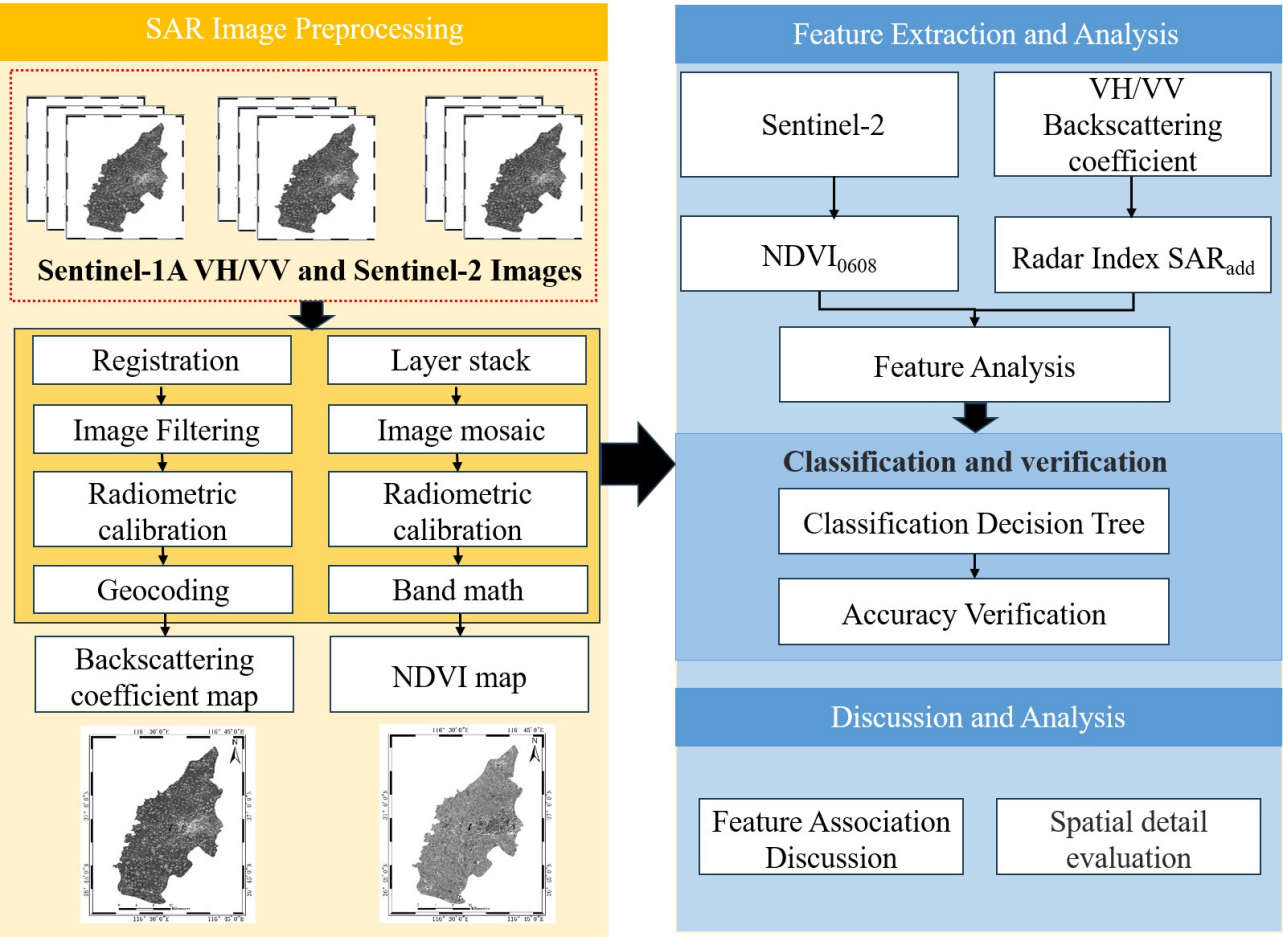
Technical flowchart. It mainly includes data preprocessing, feature extraction and feature discussion and analysis.

To extract the distribution of winter wheat, a decision tree classification method that combines mean backscattering features of time series SAR data with dual polarizations and NDVI of optical data is constructed. Based on NDVI at maturity stage, the winter wheat is distinguished from other vegetation, and then it is extracted by SAR backscattering features. The accuracy is compared with the classification results obtained using SVM classifier on multi-temporal composite SAR pseudocolor image and single Sentinel-2 optical image. Finally, the influence of decision tree constructed through various methods on the accuracy of winter wheat extraction are discussed, and the spatial details of all classification methods are evaluated qualitatively. The flowchart studied in this paper is shown in Fig 2.

### 3.1 SAR feature acquistion

#### 3.1.1 Mean backscattering feature

The SAR backscattering coefficient is mainly related to vegetation surface roughness, ground moisture, and terrain [35]. First, the dual-polarization Sentinel-1 GRD data are preprocessed as follows to obtain the backscattering characteristics:

① SAR image registration. The purpose is to remove geographical deviation. The cross-correlation method is used for registration [36].

② SAR image filtering. The purpose is to remove the influence of speckle noise on the backscattering coefficient in SAR images. The refined Lee filtering method is used [37].

③ SAR radiometric calibration. The goal is to convert the gray value (DN) of the SAR image into backscattering coefficient (dB) and the conversion Formula (1) is as follows:

$${\sigma (i,j)}_{dB}=\frac{{{DN}^{2}}_{ij}}{A^{2}}$$ (1)

The subscript *ij* refers to the position of the pixel in the image, where *i* is row and *j* is column. *A* is the radiometric calibration parameter.

④ Geocoding. The purpose is to transform the pixels from the radar coordinate system to the WGS84 geographic coordinate system. Standardization sentinel-1 and sentinel-2 images through processing.

⑤ Mean processing. Through the preceding four steps, 9 images of backscattering coefficient under VH and VV polarization are obtained. However, due to various interference and factor affecting SAR data, noise can easily be introduced. To solve this issue, the mean processing is applied to all acquired backscattering coefficient images. Using band math tool to perform mean processing on time series images. The purpose of this step is to further remove noise and reveal the mean backscattering features of different ground objects [38].

Finally, the mean backscattering coefficient images under VH and VV polarization are obtained, as shown in Fig 1(A) and 1(B). It can provide sufficient discrimination among different ground object only relying on individual backscattering coefficient *σ*_*vh*_ and *σ*_*vv*_. In order to enhance the distinction between various types of ground features, this paper referred to the polarimetric-derived parameter *SAR*_*diff*_ [39] and made some modifications to it. We construct *SAR*_*add*_ as a radar index based on the dual-polarization SAR backscattering coefficient. Comparing with *SAR*_*diff*_, *SAR*_*add*_ can widen the numerical differences between different ground objects, making it easier to establish classification thresholds. The formulated *SAR*_*add*_ equation is as follows.


$${SAR}_{add}=\sigma_{vh}+\sigma_{vv}$$ (2)

Where, *σ*_*vh*_ represents the mean backscattering coefficient under VH polarization; *σ*_*vv*_ represents the mean backscattering coefficient under VV polarization. The unit of *SAR*_*add*_ is dB.

**Fig 3:**
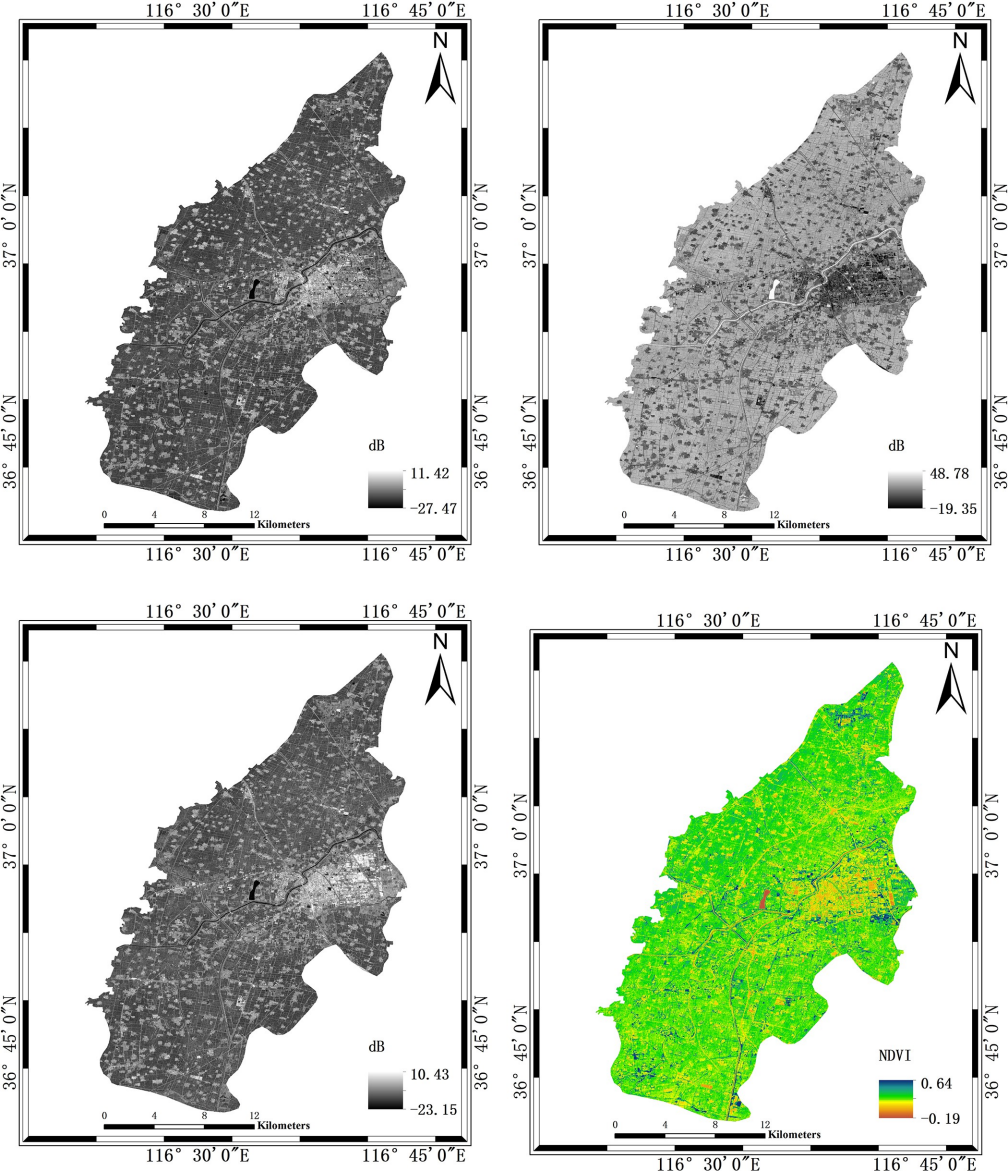
Feature map. (a) is an average backscattering coefficient map under VH polarization over multiple periods; (b) is an average backscattering coefficient map under VV polarization over multiple periods; (c) is the *SAR*_*add*_ map according to the Formula (2) and (d) is the NDVI map at maturity. All the legends are marked in the lower right of the figures.

According to the established radar index, the *SAR*_*add*_ is calculated based on the mean backscattering coefficient maps under VH and VV polarization, as shown in Fig 3(C).

### 3.2 Optical feature acquistion

The NDVI is currently the best indicator for monitoring vegetation growth status, with values ranging between -1 and 1 [12]. In the process of designing the decision tree, a single Sentinel-2 image is combined. The image at the maturity of winter wheat is considered to be the best image for distinguishing winter wheat [15], so the image with the imaging date of June 8, 2022, is selected to extract the NDVI, as shown in Fig 3(D). Since a single image could not cover the entire study area, the same period of images are mosaicked into an image that would cover the entire study area. The acquisition dates of the images are the same. Then the study area is cropped from that image.

The formula for calculating the NDVI is as follows [12]:

$$NDVI=\frac{B8-B4}{B8+B4}$$ (3)

Where, B8 represents the reflectance in the near-infrared band, and B4 represents the reflectance in the red band. B8 and B4 are all bands of Sentinel-2 image.

### 3.3 Analysis of eigenvalues of different ground object

**Fig 4:**
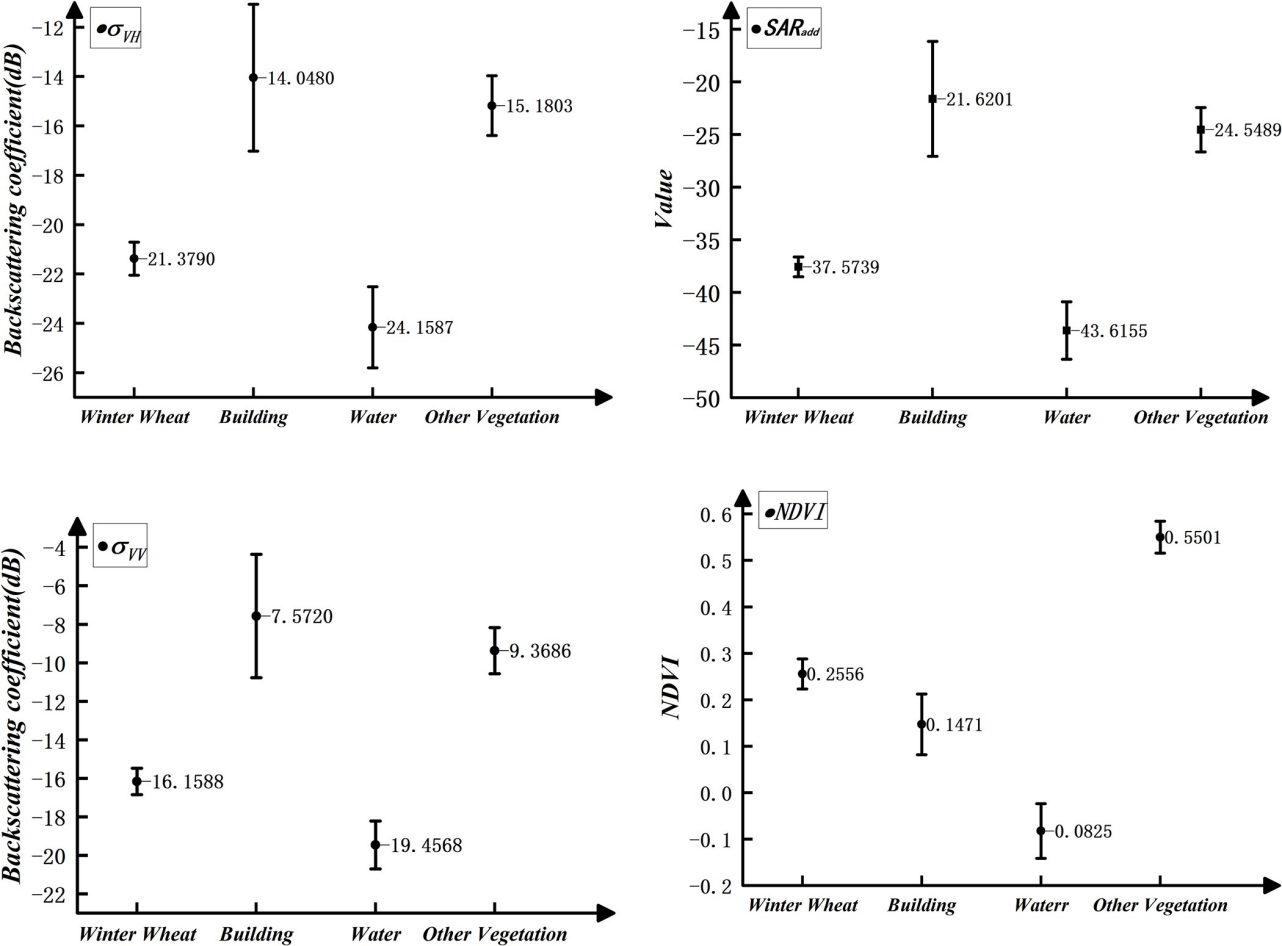
Statistical map of the mean values of the four classes. (a) is the mean and the deviation of backscattering coefficient under VH polarization; (b) is the mean and the deviation of backscattering coefficient under VV polarization; (c) is the mean and the deviation of radar index *SAR*_*add*_; (d) is the mean and deviation of the NDVI at the maturing. The numbers in the figure (a), (b) and (c) represent backscattering coefficient. The numbers in the figure (d) represent NDVI.

The eigenvalues can reflect the characteristics of ground object in study area. Based on the samples, the mean values of the backscattering coefficient, the constructed radar index, and the NDVI for all ROIs of the same samples are calculated. All eigenvalues are assessed by ENVI software. The statistical *σ*_*vh*_, *σ*_*vv*_, *SAR*_*add*_ and *NDVI*_*maturity*_ for the four ground objects are obtained, as shown in Fig 4(A)–4(D).

In Fig 4(A) and 4(B), winter wheat has small variance and is well distinguished from other ground object, which proves that it is feasible to extract winter wheat by SAR backscattering feature. However, the values of winter wheat and water are relatively close, and there is the possibility of abnormal pixel confusion. Although the difference is relatively obvious, the values are closer to those of water and other vegetation. In Fig 4(C), under the radar index *SAR*_*add*_, the value of water is less than -40dB. The variance of winter wheat is further reduced, and the differences with water and other ground object are enlarged. Therefore, using *SAR*_*add*_ can solve the confusion of winter wheat and water caused by using single backscattering feature. However, it has certain limitation to solely rely on the *SAR*_*add*_ for classification in the decision process. The median-based segmentation method introduces a certain level of randomness when relying on a single indicator. This could potentially lead to inaccurate classification results. The introduction of the NDVI can collaboratively achieve accurate extraction of winter wheat. Based on the *SAR*_*add*_ feature, water can be better extracted. Under the NDVI of winter wheat at maturity, the NDVI values of other vegetation are much higher than winter wheat. At this time, the differentiation between winter wheat and other vegetation is the highest, as shown in Fig 4(D). This difference is also significantly greater than the use of SAR backscattering feature. By introducing NDVI into the decision tree for preliminary classification, the confusion caused by SAR backscattering feature can be further reduced.

### 3.4 Establishing the decision tree

**Fig 5:**
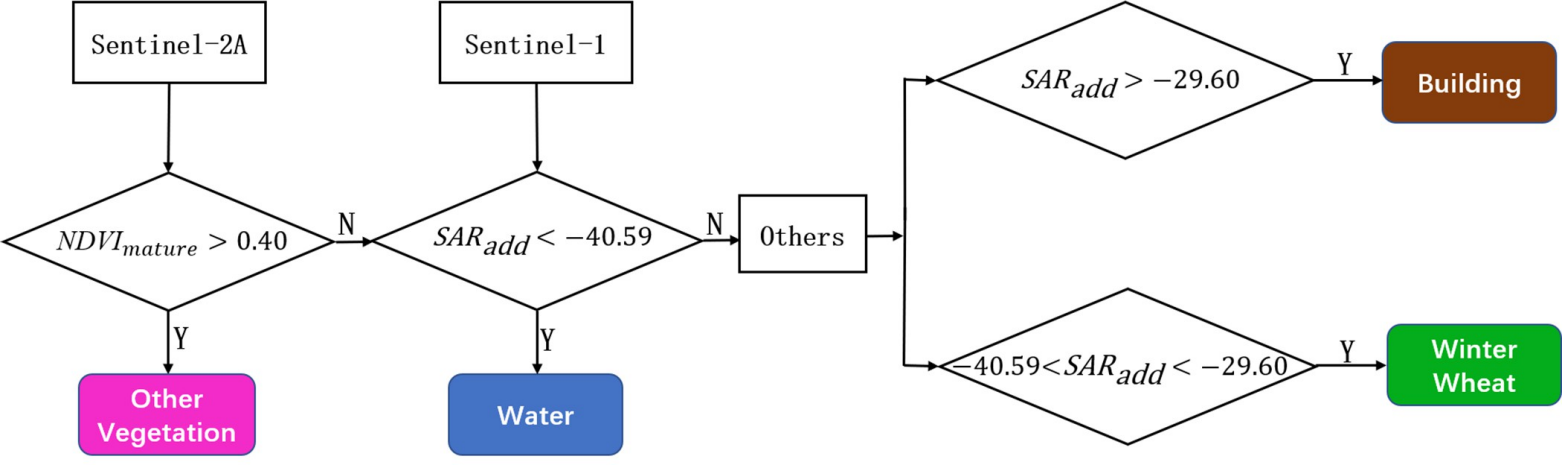
Decision tree constructed for extracting the winter wheat. The black diamond box indicates the setting of the NDVI threshold extracted at maturity and the constructed radar index *SAR*_*add*_ threshold, respectively. Together, they complete the classification of the ground objects.

According to the sample data selected in this paper, we adopt the median method to divide the threshold of constructing features and takes the median value of the statistical mean of each two classes to be distinguished. Finally, a new decision tree is constructed to extract the winter wheat, as shown in Fig 5. Because NDVI distinguishes winter wheat and other vegetation best, NDVI is firstly used to extract other vegetation, using the median of the statistical mean of other vegetation and winter wheat as the threshold. When *NDVI*_*maturity*_ > 0.40, the ground object is classified as other vegetation. In the same way the remaining classes are differentiated into water, winter wheat, and building by using the *SAR*_*add*_ feature. The criteria of the decision tree are as follows: When *SAR*_*add*_ < -40.59, the ground object is classified as water. When -40.59 < *SAR*_*add*_ < -29.60, the ground object is classified as winter wheat. When *SAR*_*add*_ > -29.60, the ground object is classified as building. Following the above principles, the final decision tree used for extracting the winter wheat in this study is established.

### 3.5 Contrast method and precision evaluation index

**Fig 6:**
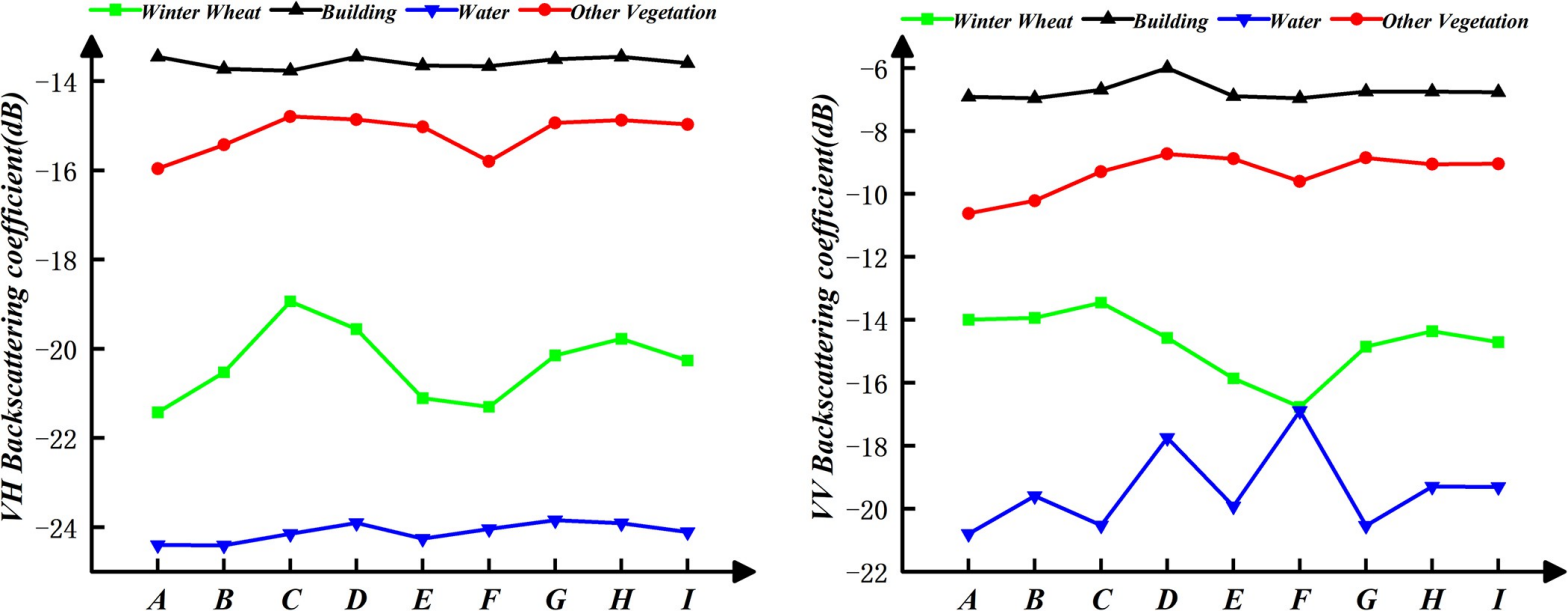
Temporal curves. A, B, C, D, E, F, G, H, I represent regreening, erecting, jointing, booting, heading, flowering, seeding, waxing, maturing of the winter wheat, respectively. (a) and (b) represent the temporal backscattering coefficient curves under VH and VV polarizations, respectively.

It has been significant method to use image color differences for supervised classification in the field of SAR remote sensing [38]. To verify the advantages of combining *SAR*_*add*_ feature and NDVI feature, we compare the classification extraction results of SVM classifier using synthesizing the pseudocolor image with the decision tree method constructed in this paper. We use a total of 9 Sentinel-1A GRD images under VH and VV polarization in this study, covering the main growth stages from regreening to maturing of winter wheat. Simultaneously, based on the statistical values of the sample data in this paper, we use it to describe time series feature curves for each class. These curves are shown in Fig 6(A) and 6(B). In the statistics of backscattering coefficient of different classes, it is analyzed that the backscattering coefficient of the different classes varied greatly at the booting, heading and flowering of winter wheat. Because there are significant differences during these three periods. The channels in periods of booting, heading and flowering are selected for synthesizing the pseudocolor image to achieve better classification. The channels are R (VH backscattering on March 31, 2022), G (VH backscattering on April 24, 2022) and B (VH backscattering on May 06, 2022).

In addition, we also compared the winter wheat extraction results of decision tree with the extraction results by optical image. The J-M distance is an effective measure for assessing the separability of training samples in remote sensing classification [40]. Based on the results of J-M distance calculations, an optical image with the highest degree of distinctiveness is chosen for classification. Finally, we select the optical image which aquired on May 4, 2022 to perform classification. Because it’s J-M distances are all above 1.9 as shown in Table 3.

**Table 3:** Results of J-M distance.

Classes	Winter Wheat	Building	Water	Other Vegetation
**Winter Wheat**	1	2.00	2.00	1.99
**Building**	2.00	1	2.00	1.99
**Water**	2.00	2.00	1	2.00
**Other Vegetation**	1.99	1.99	2.00	1

**Fig 7:**
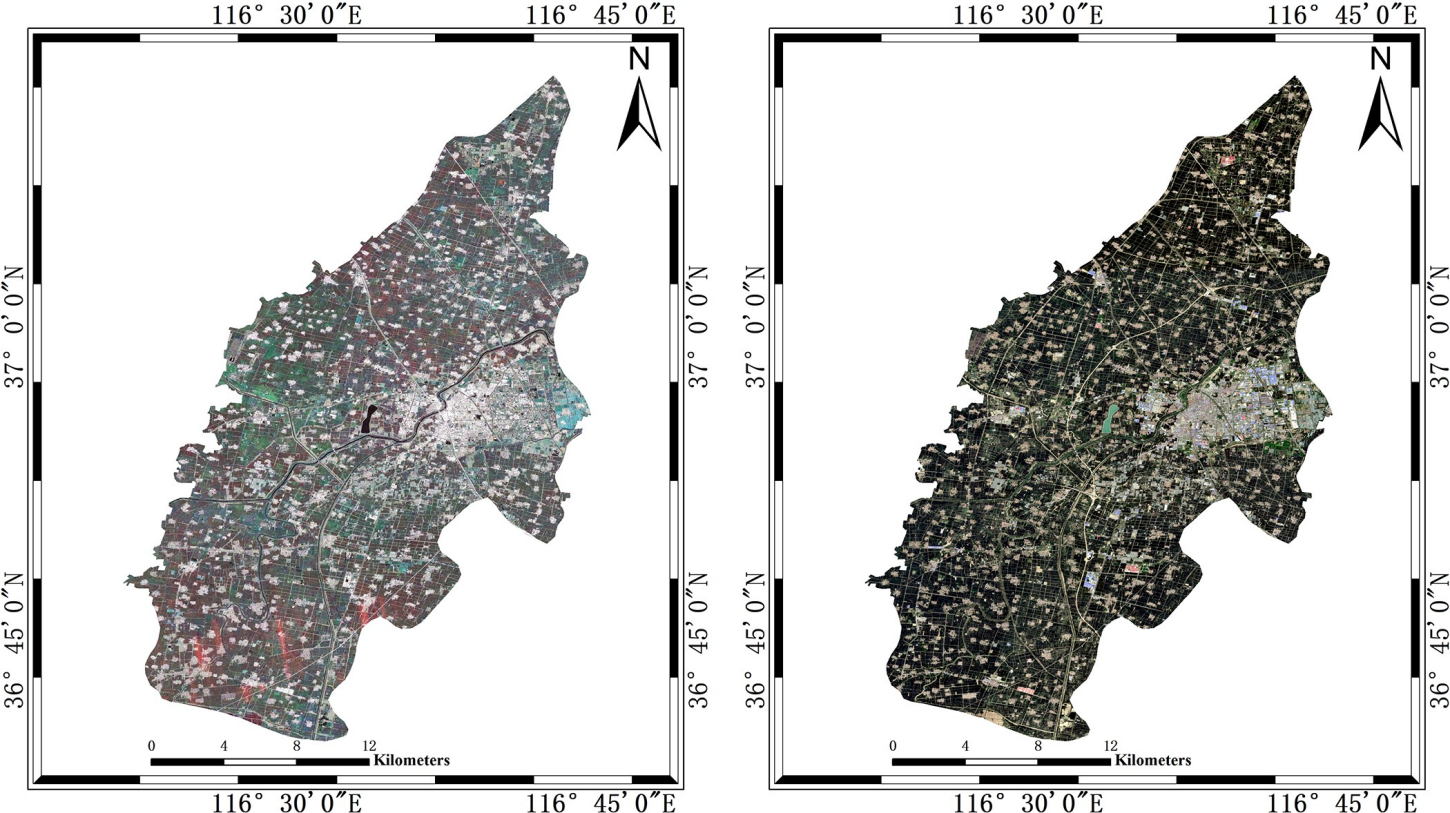
SAR and optical image for classification. (a) is the muiti-temporal composite SAR pseudocolor image. The channels are R (VH backscattering on March 31, 2022), G (VH backscattering on April 24, 2022), and B (VH backscattering on May 06, 2022). (b) is the Sentinel-2 image which aquired on May 4, 2022. It is composed of B1, B2 and B4.

SVM as a classical machine learning classifier, is a linear classifier for binary classification of data, and its decision boundary is the maximum margin hyperplane solved for the samples, which is widely used in winter wheat extraction and it achieved good results [41]. Therefore, the method of decision tree proposed in this paper is compared with the results of using the SVM classifier on multi-temporal composite SAR pseudocolor image and single Sentinel-2 image. The multi-temporal composite SAR pseudocolor image and optical image are shown in Fig 7(A) and 7(B).

The accuracy evaluation metrics used in this study mainly include Overall accuracy (OA) [42], Kappa coefficient (Kappa) [43], Producer accuracy (PA) [44] and User accuracy (UA) [44].

The OA is calculated as follows [42]:

$$OA=\frac{TP+TN}{TP+FN+FT+PN}$$ (4)

Where TP is the positive samples correctly classified by the method; FN is the positive samples incorrectly classified by the method; FP is the negative samples incorrectly classified by the method; TN is the negative samples correctly classified by the method. OA is the ratio of the number of correctly classified samples to the number of all samples.

The Kappa coefficient is calculated as follows [43]:

$$kappa=\frac{p_{0}-p_{e}}{1-p_{e}}$$ (5)

where *p*_0_ is the sum of the number of correctly classified samples in each category divided by the total number of samples, which is the overall classification accuracy. Assuming a total sample format of n, *p*_*e*_ is calculated as shown below:

$$p_{e}=\frac{\sum_{i=1}^{C}a_{i}\times b_{i}}{n\times n}$$ (6)

where *a*_*i*_ is the number of true samples in each category, and the number of predicted samples in each category is *b*_*i*_.

The PA is calculated as follows [44]:

$$PA=\frac{TP}{TP+FN}$$ (7)

The UA is calculated as follows [44]:

$$UA=\frac{TP}{TP+FP}$$ (8)

## 4 Results and discussion

### 4.1 Classification results

**Fig 8:**
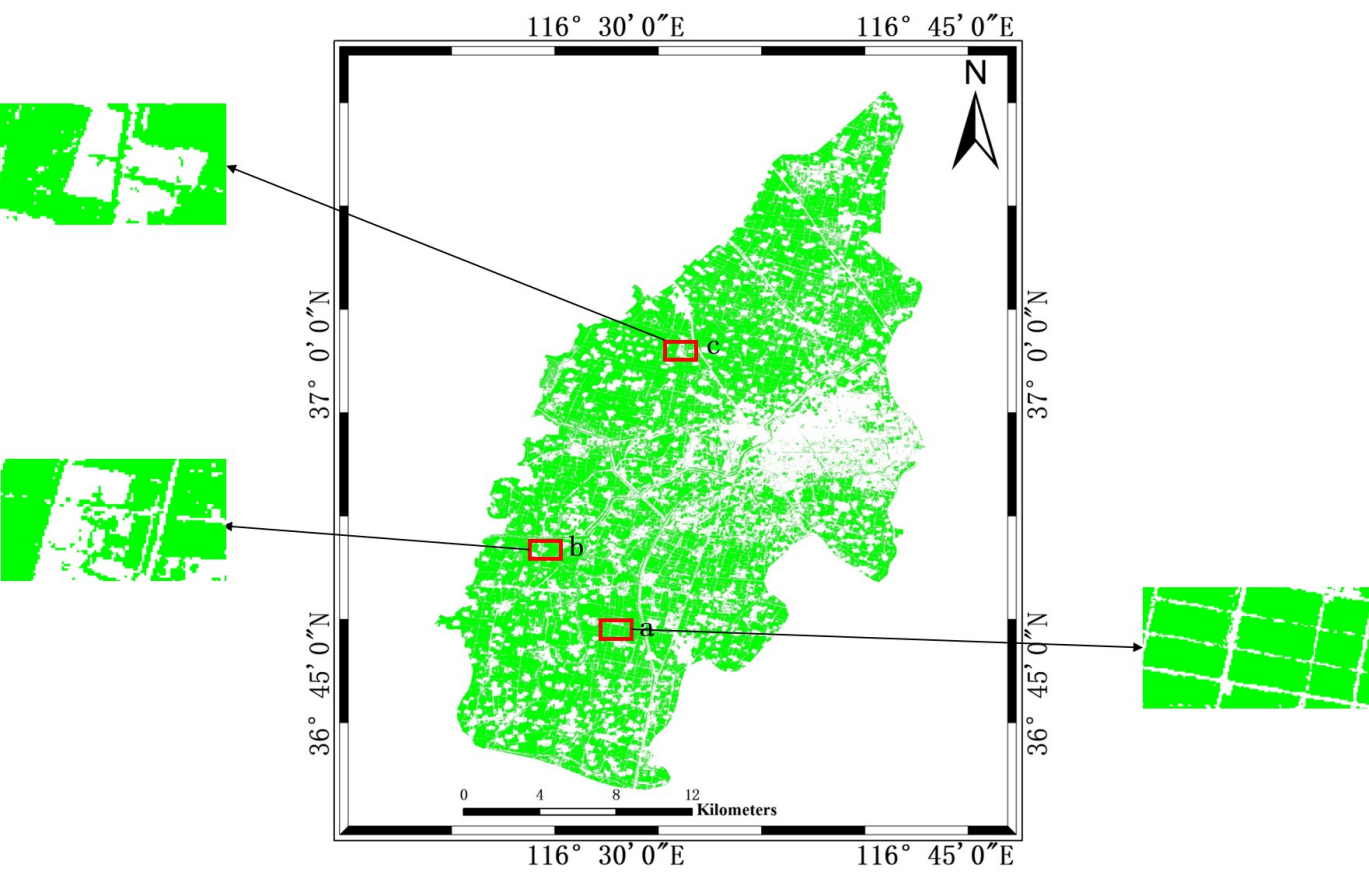
Decision tree classification results and zoomed-in regions a, b, c.

The distribution of winter wheat in 2022 in the study area is mapped using the decision tree model proposed in this paper, as shown in Fig 8. In the classification results, the winter wheat nearby the city is relatively scattered, while the winter wheat of the other areas is uniformly distributed. Concerning the research results of related scholars [45], the distribution of winter wheat is basically consistent with the results of our paper, the results of the decision tree extraction in this paper are basically accurate. Meanwhile, three small regions (a, b, c) are randomly selected in the classification results of this paper to demonstrate the difference in details with other classification schemes and features. All the comparisons are shown in Fig 12.

**Fig 9:**
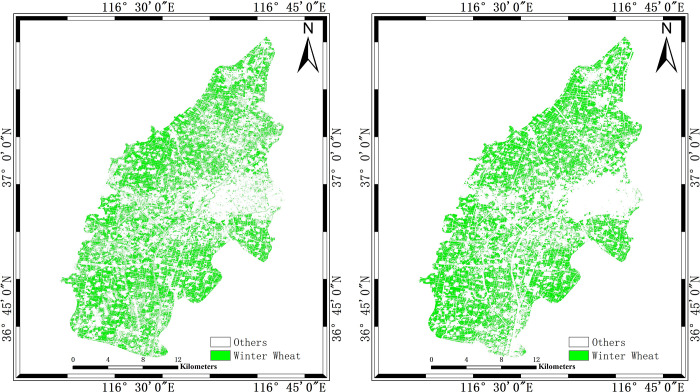
SVM classification results. (a) is the classification result from multi-temporal composite SAR pseudocolor image, (b) is the classification result from single Sentinel-2 image.

**Fig 10:**
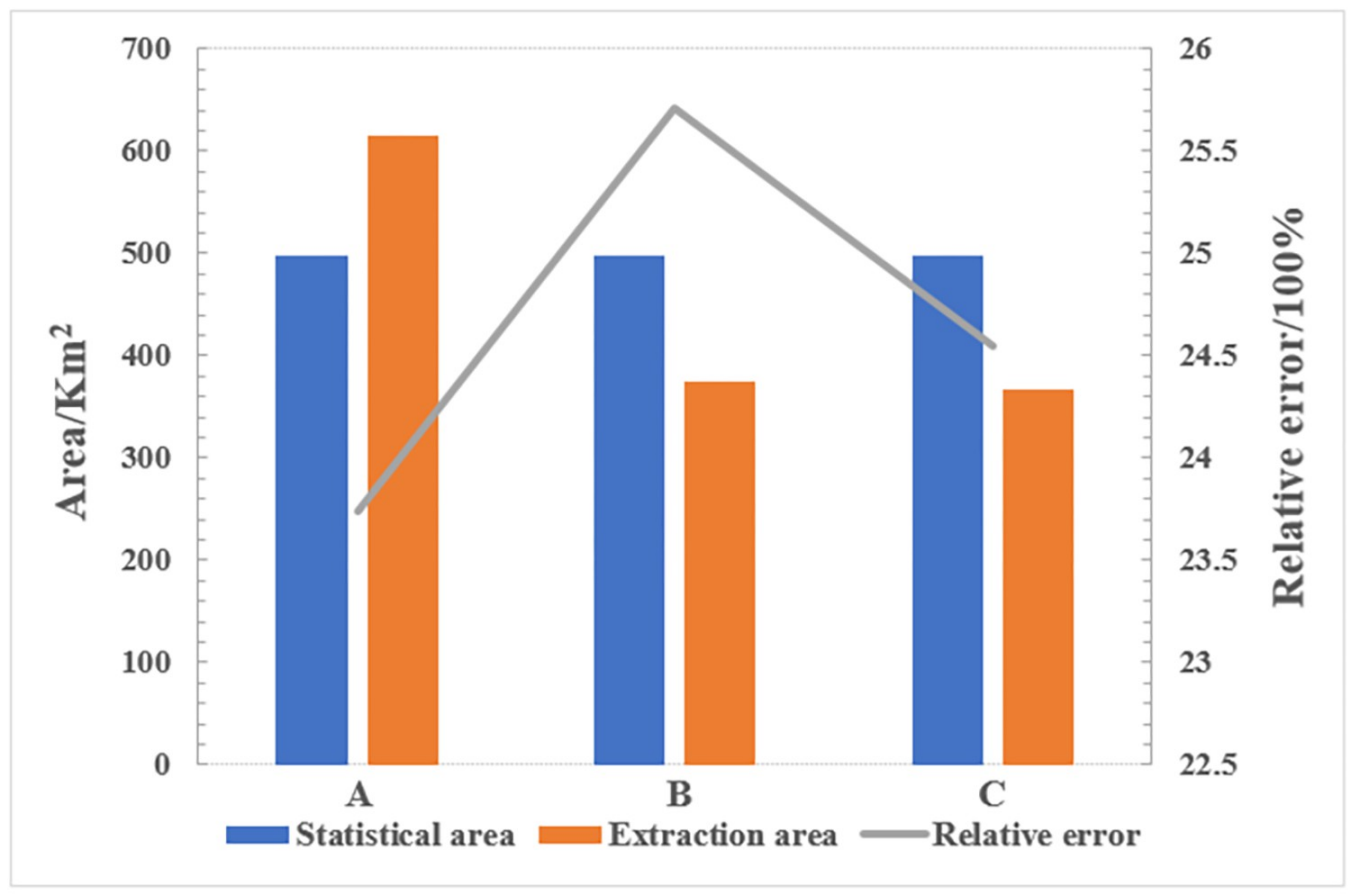
The extraction area of winter wheat and relative error with official statistics data. (a), (b) and (c) indicate the classification result of the decision tree method of this paper, Single Sentinel-2 image and Multi-temporal composite SAR pseudocolor image, respectively.

The classification results of this paper are compared with the classification results of multi-temporal composite SAR pseudocolor image and single Sentinel-2 image by SVM classifier as shown in Fig 9(A) and 9(B). The accuracies of the extraction results of the three methods are shown in Table 4. The extraction area of winter wheat and relative error with official statistics data are shown in Fig 10.

**Table 4:** Classification accuracy of different classification methods.

Method	(a)The decision tree method of this paper (our method)	(b)The SVM method use single Sentinel-2 image	(c)The SVM method use multi-temporal composite SAR pseudocolor image
**winter wheat PA**	93.32%	80.17%	73.30%
**winter wheat UA**	97.45%	96.82%	92.98%
**OA**	96.10%	90.44%	85.41%
**Kappa**	0.94	0.86	0.78

In Fig 9, we can observe that the winter wheat extraction results of using the SVM based on multi-temporal composite SAR pseudocolor image and single Sentinel-2 image method are fragmented near the urban area of the research area. In comparison, the decision tree method proposed in this article has significant improvements in this regard.

In Table 4, only the PA and UA of the decision tree method proposed in this paper can reach 90% when compared with the results of multi-temporal composite SAR pseudocolor image and single Sentinel-2 image using the SVM. The OA and Kappa of the method proposed in this study are 96.10% and 0.94, respectively. Compared with the results from the multi-temporal composite SAR pseudocolor image and single Sentinel-2 image using the SVM classifier, the OA is improved by 10.69% and 5.66%, and the Kappa is improved by 0.16 and 0.08, respectively.

Based on Fig 10, the best extraction result is the decision tree method proposed in this paper. We also calculate the planting area and relative error of winter wheat. The relative errors of the decision tree method of this paper are the lowest, with values around 23.74%. The relative errors of other two classification results are 25.71% and 24.55%. This paper adopts a method combining time series dual-polarization SAR features and NDVI, demonstrating superior performance and applicability in winter wheat extraction. By employing machine learning algorithms, it enhances accuracy and reliability in winter wheat extraction. Targeting characteristics of winter wheat growth stages, tailored feature extraction and classification strategies significantly improve wheat extraction effectiveness. Compared to other method, this method exhibits superior capability and accuracy in distinguishing winter wheat, showcasing enhanced performance and precision. In contrast, the method of the decision tree proposed in this paper is more suitable for extracting the winter wheat.

### 4.2 Classification results discussion of feature component contributions

**Fig 11:**
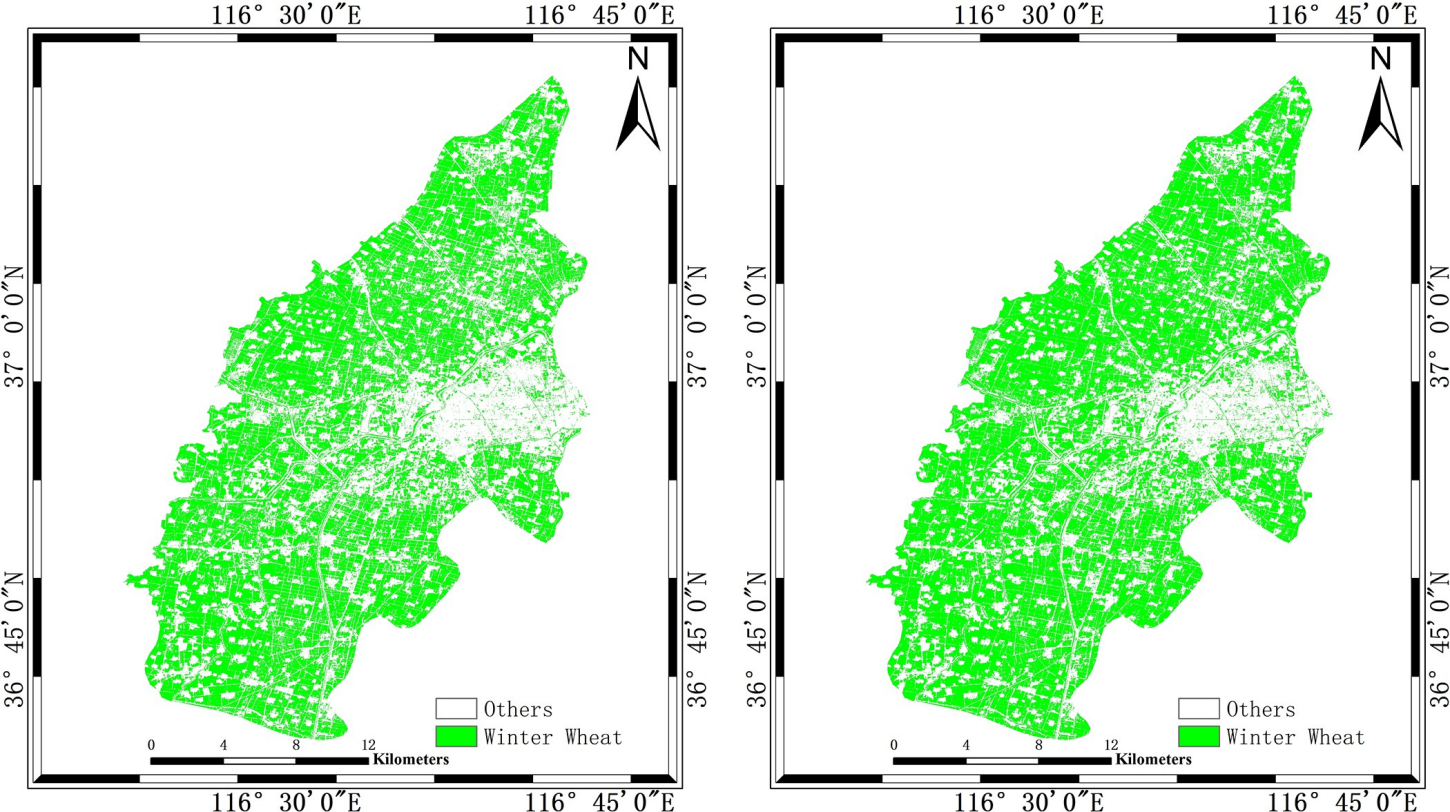
Classification results with different features. (a) is the classification result only using *SAR*_*add*_ feature. (b) is the classification result using both *SAR*_*add*_ feature and NDVI.

To quantitatively explore the role of features and the advantages of combing SAR image and optical image, two decision tree classification schemes are constructed, as shown in Table 5. There are two classification schemes: one involves incorporating *SAR*_*add*_ feature exclusively, the other incorporates both *SAR*_*add*_ feature and NDVI. The decision tree classification scheme1 only use *SAR*_*add*_ feature to adopt the median value of the statistical mean of each two classes. The decision tree classification scheme2 introduces *NDVI*_*maturity*_ on the basis of scheme1. Then the scheme2 also adopt the median value of the statistical mean of each two classes. The decision tree classification results are shown in Fig 11(A) and 11(B). We can find that the extraction effect of winter wheat is better when both *SAR*_*add*_ and *NDVI*_*maturity*_ are used simultaneously, and the extraction results of winter wheat near the urban area of the study area are more continuous.

**Table 5:** Classification scheme and accuracy.

Scheme	Feature	PA	UA
**Scheme1**	*SAR*_*add*_	90.24%	89.29%
**Scheme2 (our method)**	*SAR*_*add*_+*NDVI*_*maturity*_	93.32%	97.45%

According to the classification results and the validation samples of this paper, the PA and UA of the two decision tree classification schemes are obtained. In Table 5, when both the *SAR*_*add*_ feature and NDVI were introduced simultaneously, the PA and UA for extracting the winter wheat are higher by 3.08% and 8.16% respectively than those of using only the *SAR*_*add*_ feature. When the *SAR*_*add*_ feature and NDVI were introduced, the winter wheat PA is improved by 3.08% and the UA is improved by 8.25%.

**Fig 12:**
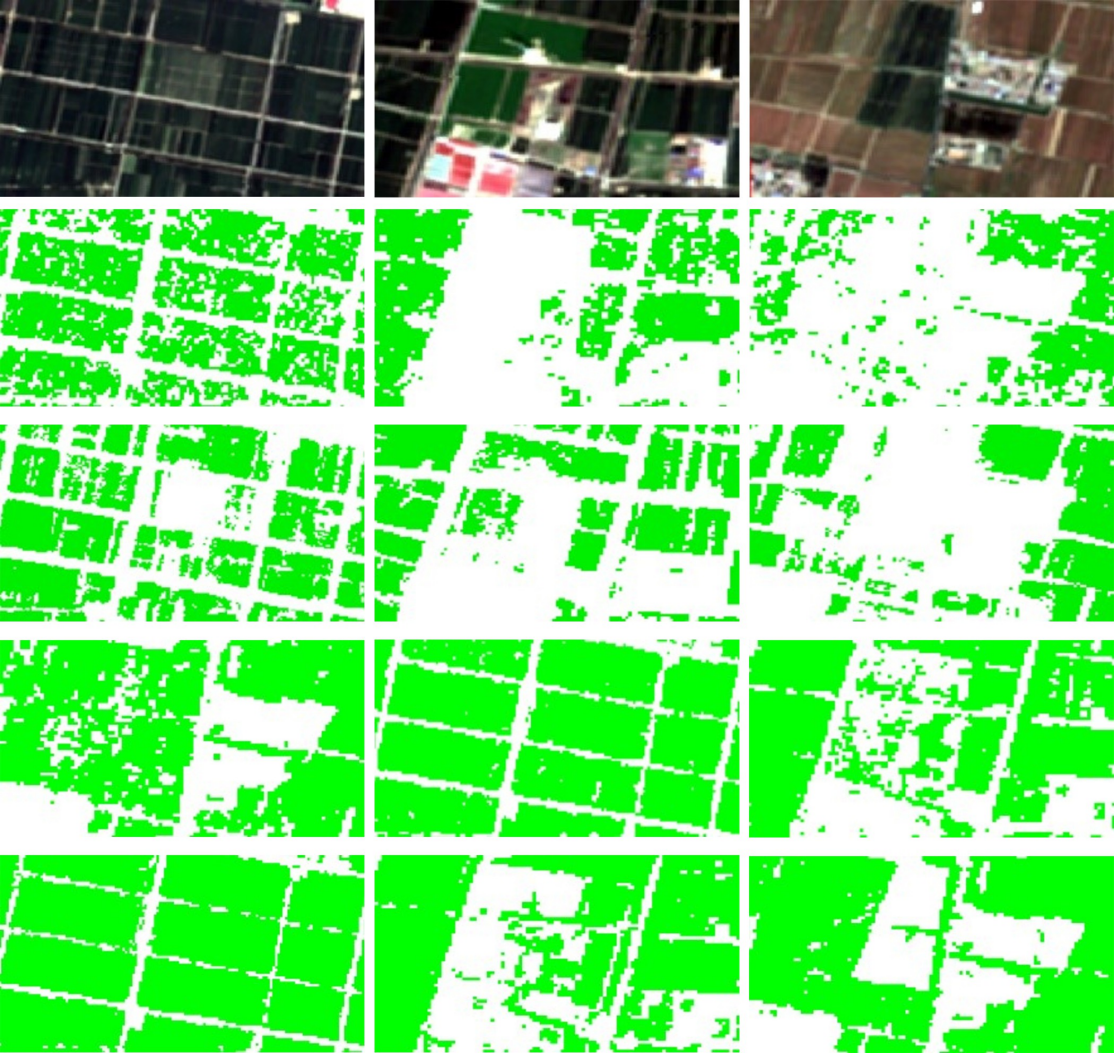
Comparison of spatial details. (a) is the result from Sentinel-2 RGB image. (b) is the result from multi-temporal composite SAR pseudocolor image. (c) is the result from single Sentinel-2 image. (d) is the classification result of scheme1. (e) is the classification result of scheme 2. Region a, Region b, Region c are randomly selected subregions used to demonstrate the advantages of the method in this paper.

Combining the winter wheat distribution results extracted using SVM classifier from the previous section, this paper presents the localized zoom region results of winter wheat using Sentinel-2 RGB image, single Sentinel-2 image, multi-temporal composite SAR pseudocolor image, Scheme1 and Scheme2, as shown in Fig 12.

Comparing the above extraction results of winter wheat, it is easy to find that the classification results from multi-temporal composite SAR pseudocolor image and single Sentinel-2 image are likely to be fragmented, which leads to missed classification. And these two methods are also relatively fragmented in extracting ground objects. This leads to inaccurate extraction area of winter wheat. This phenomenon is significantly improved in the method proposed in this paper. In addition, comparing the classification Scheme 1 (Sentinel-1 single image) and Scheme 2 (Sentinel-1 and 2 images), it is easy to find that the other vegetation is often misclassified as winter wheat when only use SAR feature to perform classification. As shown in Scheme 1. In contrast, the method proposed in this paper significantly avoids the occurrence of such problem by combining time series SAR features and NDVI. This increases the extraction accuracy of winter wheat. In summary, the decision tree method for extracting the winter wheat proposed in this paper makes up for the respective deficiencies of SAR and optical data and has outstanding advantages.

## 5 Conclusions

In this paper, combining the advantage of SAR data and optical data, a decision tree methodology that integrates time series dual-polarization mean backscattering coefficient and NDVI is constructed to extract the winter wheat, and contrast comparative experiments are carried out to evaluate the performances of the proposed method. Meanwhile, the classification results of feature component contributions are discussed and explored. We can draw some valuable conclusions as follows:

1. The OA and Kappa coefficient of the method proposed in this paper are 96.10% and 0.94, respectively. Compared with the result from multi-temporal composite SAR pseudocolor image classification by SVM classifier, the OA and the Kappa are improved by 10.69% and 0.16, respectively. Compared with the result from single Sentienl-2 classification by SVM classifier, the OA and the Kappa are improved by 5.66% and 0.08, respectively. This shows that the method proposed in this paper is more accurate and more suitable for extracting the winter wheat.
2. It can effectively improve classification accuracy combining time series SAR feature with optical feature. When only using the SAR feature for decision tree classification, the PA and UA of winter wheat are the lowest at 90.24% and 89.20%, respectively. With the introduction of the optical feature NDVI, the PA and UA of winter wheat improved by 3.08% and 8.25%, reaching 93.32% and 97.45%, respectively. By introducing the NDVI of the maturity stage, the confusion problem of SAR backscattering feature during the extraction of winter wheat is effectively reduced.

In this paper, the SAR data is the main data, and the optical data is the auxiliary data. By combining the time series SAR features and NDVI, the classification results with higher accuracy are obtained than those only using SAR data or optical data. It provides a theoretical basis for extracting the winter wheat more precise. However, the number of features considered in this paper is still relatively limited. In the future we will explore the potential of more multi-polarization features, texture features, and other vegetation index features in the extraction process of winter wheat.
